# Targeting Collagen Pathways as an HFpEF Therapeutic Strategy

**DOI:** 10.3390/jcm12185862

**Published:** 2023-09-09

**Authors:** Alice Bonanni, Ramona Vinci, Alessia d’Aiello, Maria Chiara Grimaldi, Marianna Di Sario, Dalila Tarquini, Luca Proto, Anna Severino, Daniela Pedicino, Giovanna Liuzzo

**Affiliations:** 1 Department of Cardiovascular Sciences, Fondazione Policlinico Universitario A. Gemelli IRCCS, 00168 Rome, Italy; alice.bonanni@guest.policlinicogemelli.it (A.B.); dalila.tarquini@guest.policlinicogemelli.it (D.T.); luca.proto01@icatt.it (L.P.); daniela.pedicino@policlinicogemelli.it (D.P.); giovanna.liuzzo@unicatt.it (G.L.); 2 Department of Cardiovascular and Pneumological Sciences, Catholic University of Sacred Heart, 00168 Rome, Italy; ramona.vinci@unicatt.it (R.V.); mariachiara.grimaldi@unicatt.it (M.C.G.); anna.severino1@unicatt.it (A.S.); 3 Department of Anaesthesia and Intensive Care, IRCCS Istituto Clinico Humanitas, Humanitas University, 20089 Milan, Italy; mariannadisario@gmail.com

**Keywords:** HFpEF, coronary microvascular dysfunction, cardiac remodeling, collagen, SGLT2i, AGEs

## Abstract

Heart failure with preserved ejection fraction (HFpEF) is a complex and heterogeneous clinical syndrome. The prevalence is expected to increase in the coming years, resulting in heart failure with reduced ejection fraction (HFrEF). This condition poses a burden to the global health care system as the number of patients affected by this condition is constantly increasing due to a rising average lifespan. The absence of validated drugs effective in reducing hospitalization rates and mortality may reflect the impossibility of applying a one size fits all approach as in HFrEF, heading for a personalized approach. Available evidence demonstrated the link between collagen quantity and quality alterations, and cardiac remodeling. In the context of fibrosis, collagen cross-linking is strictly involved, displaying two types of mechanisms: enzymatic and non-enzymatic. In the murine model, enzymatic inhibition of fibrosis-inducing protease-activated receptor-1 (PAR1) and transforming growth factor (TGF)-β signaling appeared to reduce cardiac fibrosis. On the other hand, in the case of non-enzymatic cross-linking, sodium glucose co-transporter type 2 inhibitors (SGLT2is), appeared to counteract the deposition of advanced glycation end-products (AGEs), which in turn contributed to ventricular remodeling. In this review, we address the mechanisms associated with collagen alterations to identify potential targets of cardiac fibrosis in HFpEF patients.

## 1. Introduction

Heart failure with preserved ejection fraction (HFpEF) is a complex and heterogeneous clinical syndrome that results from heart structural and functional changes, which makes the heart able to adequately pump blood to peripheral districts only at the cost of elevated cardiac filling pressures. This syndrome is characterized by signs and symptoms of HF, such as dyspnea and fatigue, but with a normal left ventricular ejection fraction (LVEF) and, evidence of structural and/or functional cardiac abnormalities consistent with the presence of LV diastolic dysfunction and raised LV filling pressures, according to the latest European Society of Cardiology guidelines [1]. More often diagnosed in women than in men, HFpEF is defined as a “female pattern”, even though it may soon be overcome because of the increasing diagnoses in men [2]. Due to its heterogeneous etiology, HFpEF embraces different phenotypes, sometimes showing a more fibrotic biological profile, while, on other occasions, displaying a more marked inflammatory identity [3]. This condition represents a burden on the health care system worldwide as the number of HFpEF patients is steadily increasing—firstly, due to a rise in the average life expectancy, and secondly, thanks to an easier clinical recognition—remaining one of the most challenging hot topics in the cardiovascular field. Indeed, because of its nature, the scientific community still lacks a perfect experimental animal model that mimics this complex condition, making drug development harder. The absence of effective drugs in reducing hospitalization rates and mortality may reflect the difficulty of applying a one size fits all approach like in HFrEF. Furthermore, studies have shown that a more targeted and personalized approach seems more appropriate [4]. Diagnostic work-up in HFpEF involves symptoms and signs of HF, LVEF ≥ 50%, elevated natriuretic peptides and, at least one of the additional criteria among relevant structural heart disease assessed by echocardiographic examination (left ventricular hypertrophy, LVH and left atrial enlargement, LAE) and diastolic dysfunction [1]. Indeed, recent studies have raised the matter of the diagnostic utility of the echocardiographic parameter represented by the E/e′ ratio (ratio of the early diastolic velocity of mitral inflow to the early diastolic velocity of mitral annular motion (e′)), extensively used as a non-invasive surrogate for LV filling pressures, for the diagnosis of HFpEF [5]. Nevertheless, alternative echocardiographic parameters, as proposed in the 2021 European Society of Cardiology Guidelines [1], should be considered to clarify the diagnostic work-up of HFpEF. In particular, the LV mass index, relative wall thickness, left atrium (LA) volume index and pulmonary artery (PA) systolic pressure have gained great interest in recent consensus [6]. Indeed, in HFpEF, misdiagnosis might stem from a multifaceted nature as well as from the reductive use of the echocardiographic E/e′ ratio itself.

## 2. Endothelial Dysfunction and the HFpEF Microvascular Paradigm

Endothelial dysfunction plays a pivotal role in the pathophysiology of HFpEF [7]. Cardiovascular comorbidities, such as hypertension, atrial fibrillation, coronary artery disease, dyslipidemia, diabetes mellitus, obesity and obstructive sleep apnea, trigger a low-grade systemic pro-inflammatory state that leads to coronary microvascular dysfunction (CMD) and affects the perivascular environment, causing fibrosis and remodeling of the heart. 

The latest updates in biomarker selection highlight the enormous complexity of CMD molecular pathways [8]. Among non-cardiovascular comorbidities, obesity elicits a chronic, low-grade systemic inflammatory state in HFpEF patients, which contributes to CMD through adipokine release and β-adrenergic receptor activation. Furthermore, obesity could be an indirect goal to stimulate other comorbidities, such as insulin resistance [9]. Diabetes mellitus also fuels the systemic inflammatory milieu, contributing to endothelial impairment. As in patients presenting with myocardial infarction [10], the concomitant metabolic dysregulation is characterized by an increase in glucose uptake and related glucose transporter 1 (GLUT-1). In the murine model, this condition is also typical of adverse LV remodeling, cardiomyocyte hypertrophy, and eventually a failing heart [11]. Hyperglycemia induces the glycation of interstitial proteins and oxidative stress leads to the formation of advanced glycation end products (AGEs), responsible for the increased LV stiffness. The transition from glucose utilization to the fatty acids-dependent state, lipotoxicity, and impaired insulin signaling, all contribute to HF in the diabetic setting [12,13].

CMD arises from a merge of elements such as vascular smooth cell overactivity, vascular remodeling and capillary rarefaction, resulting from a discrepancy between vessel destruction and vessel neoformation. Indeed, studies performed on LV myocardial autopsy specimens harvested from patients with HFpEF showed how fibrosis was inversely associated with microvascular density, but not linked to any epicardial lesion [7]. The most endorsed theory for the pathophysiological mechanism of HFpEF identifies CMD as the main driver of HFpEF development, overcoming the previous “pressure overload” model [14]. According to the CMD and inflammation hypothesis [15], an imbalance of the paracrine signaling and the microvascular permeability occurs. Endothelial inflammation induces the production of reactive oxygen species (ROS) which leads to a reduction of nitric oxide (NOx) bioavailability, and consequently to a reduction of protein kinase G (PKG); this modification promotes cardiomyocyte hypertrophy. Moreover, pro-inflammatory stimuli induce adhesion molecule expression, which, in turn, recruit monocytes. The subsequent release of transforming growth factor beta (TGF-β) engenders conversion of fibroblasts to myofibroblasts and collagen deposition. CMD is identified both as a prime mover and an enduring mechanism in the failing heart [16], and represents the element that corroborates the hypothesis of a different etiology among the HF phenotypes, thus allowing us to consider HFpEF as a different entity as compared to HFrEF, the main feature of which is represented by the loss of cardiomyocytes. 

## 3. Fibrosis and Extracellular Matrix Derangement in the Onset of HFpEF Stiffness

Fibrosis derives from the interplay of modifications that affect the cellular and extracellular microenvironment. The main changes concern cardiomyocyte hypertrophy, alteration of the giant sarcomeric myoprotein titin [17]—a bidirectional spring whose phosphorylation state affects cardiomyocyte passive stiffness—and excessive deposition of extracellular matrix (ECM). ECM alteration shapes a scaffold between cardiomyocytes and cardiac vessels leading to an imbalance of matrix metalloproteinases (MMPs) and tissue inhibitors of metalloproteinases (TIMPS) [18]. Furthermore, the myocardial ECM not only provides a mere mechanical support that preserves the geometry of the heart but constitutes a dynamic entity, whose turnover relies on fibroblasts which, in a pathological milieu, undergo a conversion towards collagen-producing myofibroblasts. 

MMPs are in a latent state and their activation can be induced by ROS generation, leading to the degradation of ECM components. MMP1, MMP8 and MMP13 have a high affinity for collagen, while other MMPs have tropism for elastin and proteoglycans [16]. Mainly elastin, but also laminin, glycoproteins, and various collagen types, were reported as sources of ECM matrikines. Matrikines, ECM-derived peptides, may regulate ECM synthesis and remodeling and MMP generation and activation [19]. Furthermore, the impaired removal of these fragments is involved in the activation of the immune-mediated response, fueling ECM degradation, and further contributing to the disruption of myocardial integrity [20]. Selective enzymes, such as MMP-2, MMP-7, TIMP-1 and TIMP-2 increase with age, while MMP-9 shows the opposite trend. Both MMP and TIMP protein levels are augmented and seem to be associated with diastolic impairment in aged and healthy individuals, although more studies are required to further clarify their role in age-related ECM modifications [21,22]. In this general scenario, cardiomyocyte stiffening and interstitial fibrosis, deriving from collagen deposition and degradation imbalance, lead to LV diastolic dysfunction, the major impairment in HFpEF patients. 

## 4. Qualitative and Quantitative Changes of Cardiac Collagen Fibers

**Figure 1:**
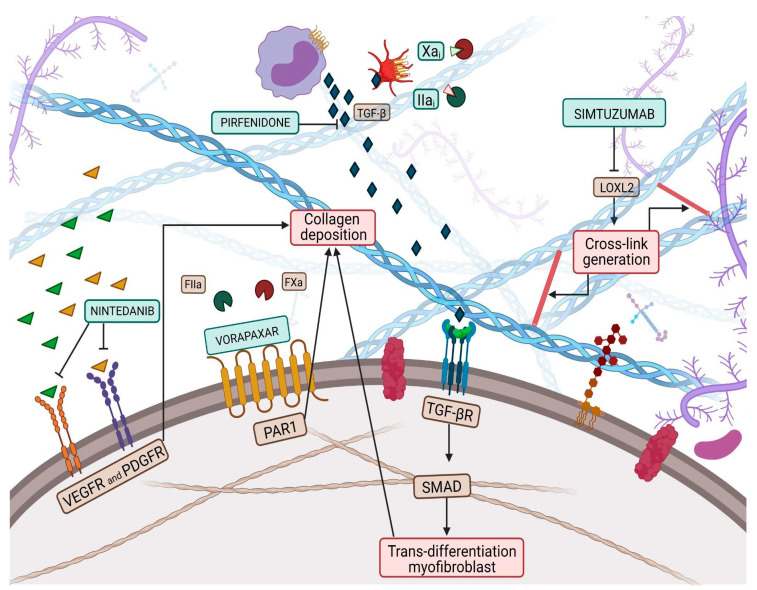
Collagen deposition and collagen cross-link (CCL) have a great influence on left ventricular (LV) stiffness in patients with heart failure (HF). CCL is the formation of intramolecular and intermolecular covalent bonds between lysine residues in collagen molecules, which greatly increases the tensile strength and stiffness of collagen fibers and makes them more resistant to degradation. Multiple pathways have been targeted by therapeutic pharmacological treatments to overcome myofibroblast production, such as nintedanib, pirfenidone, Xa and Iia inhibitors, while vorapaxar attempts to inhibit excessive collagen deposition. Finally, simtuzumab inhibits the lysyl oxidase 2 (LOX2) pathway, thus reducing collagen cross-link generation.

Collagen is a vital, life-long and highly prevalent structural protein in mammals. The collagen family embraces 28 members, each consisting of 3 polypeptide subunits that assemble into a triple-helix structure domain [23]. Fibrillary collagen type I and III are the prevailing components of the ECM, accounting for nearly 80% and 10% of collagen in the healthy heart, respectively [24]. Under physiological conditions, triple helices generate microfibrils which, once assembled together, form larger fibers, increasing tissue stability [23]. Previous studies have shown a significant sex-related alteration in collagen metabolism, especially during the menopausal and postmenopausal phases, confirming the female HFpEF pattern and period [25]. Indeed, with aging, the collagen properties change, damaging its biochemical and biomechanical characteristics [26], thus leading to the loss of flexibility and altered enzymatic digestibility [27]. Age-related alterations in collagen strands play a central role in tissue stiffening. Several studies have proven, over the years, the reduction of the collagen fiber number in elderly people [28,29]. Furthermore, elderly adult collagen net is defined by fewer, longer, and thicker fibrils than the young-adult counterpart [28], highlighting the relevance of collagen quantity and quality. Quality features, such as collagen isoforms, collagen cross-linking (CCL) degree, and collagen type I to type III ratio and solubility, also play a role in diastolic impairment [18]. In HF related to pressure overload, the ratio of collagen type I to type III is increased [30] and is due to the augmented expression of type I collagen, which leads to an imbalance between myocardial stiffness and elasticity, properties respectively provided by the collagen isoform I and isoform III. Nevertheless, in ischemic cardiomyopathy, the main cause of HFrEF, the same index appears reduced, attributable to an increased expression of type III collagen. In this perspective, the collagen type ratio together with different collagen isoforms could be exploited as intriguing tools to distinguish between the two HF phenotypes [31] and to tailor therapeutic treatment. For instance, Graziani et al. hypothesized parallelism, transposing therapeutic strategies from idiopathic pulmonary fibrosis to HFpEF, targeting collagen production, induced by transforming growth factor-beta (TGF-β) signaling pathway, adopting nintedanib and pirfenidone [32]; thus, updating the possible new applications of these pharmacological treatments (Figure 1).

## 5. Myocardial Collagen Cross-Linking and Therapeutic Involvement in the Pharmacological Treatment of HFpEF

Collagen deposition and CCL are processes that make collagen fibers more resistant to degradation and fibrosis less reversible, through the formation of intramolecular and intermolecular bonds. In particular, CCL takes place through two different mechanisms: on the one hand, through an enzymatic pathway, catalyzed by a lysyl oxidase (LOX), a copper-dependent enzyme, which acts with high affinity on lysine or hydroxylysine residues and contributes to the formation of cross-link precursor allysine aldehydes [33]. On the other hand, CCL can occur through a non-enzymatic path, via a glycation process that includes the formation and accumulation of AGEs [34]. In a physiological milieu, the time frame of these two mechanisms seems to differ: the enzymatic CCL takes place preferentially in the early stages of collagen maturation, providing for the stabilization of the collagen fibrils; while the non-enzymatic one is attributable to aging, privileging the most advanced decades [35]. 

LOX, also known as LOX1, is the progenitor of a family of enzymes responsible for collagen and elastin cross-linking, which encompasses four other members, known as LOX-like proteins (LOXL1, LOXL2, LOXL3 and LOXL4). LOX1 shares with the progenitor the secretion as a precursor form that needs to be activated through proteolysis, while the other three members of the family do not require activation, paving the way for the hypothesis that LOX1 and LOXL1 could belong to a common subfamily [36]. Evidence proved an increase in myocardial LOX expression in HFpEF patients compared to controls [37]. Furthermore, Krasner et al. observed an interesting direct correlation between myocardial LOX expression, the degree of CCL and the echocardiographic diagnostic parameter E/e’ ratio [38]. This study might have corroborated the presence of a link between increased ventricular stiffness and collagen expression in the myocardial tissue of HFpEF patients. LOX is not the only player, but LOXL proteins, and especially that of type 2, also affect CCL, contributing to impaired cardiac function. Myocardial LOXL2 appears enhanced in HFpEF patients, displaying a direct correlation with the degree of CCL and with the E/e’ ratio [39]. In light of this background, both LOX and LOXL2 could represent intriguing targets to investigate to implement the exiguous pharmacological armamentarium of HFpEF pharmacology. The inhibition of LOXL2 through the administration of a selective human monoclonal antibody, known as simtuzumab, has already been investigated in clinical trials involving patients with idiopathic pulmonary fibrosis and liver fibrosis (Figure 1) [40]. Although no clinical trials exploring the role of simtuzumab in HF are available, it has been demonstrated that antibody-mediated inhibition or genetic disruption of Loxl2 greatly reduces stress-induced cardiac fibrosis and chamber dilatation, improving systolic and diastolic functions [39]. These results pave the way for future studies in HFpEF.

However, once catalyzed, CCLs are more resistant to degradation, suggesting that a more adequate LOXL2 blockade should be performed in the earlier stages of the disease progression [39]. Despite being an attractive target, LOX inhibition could be deleterious, as its function must be preserved, in order to maintain a physiological level of CCL for the biomechanical properties of the ECM. Alternatively, Friebel et al. decided to focus on a LOXL2 target upstream of the myocardial pro-fibrotic cascade. They proposed the administration of a protease-activated receptor 1 (PAR1) inhibitor vorapaxar in mice, moving the lens on a novel and promising therapeutic approach for HFpEF, interfering with collagen quality and quantity. The choice of the upstream Fxa/FIIa-PAR1/PAR2/TGF-β axis lays the groundwork for the knowledge that activation of PAR2 is a pivotal regulator of the fibrosis-inducing PAR1 and TGF-β signaling. Furthermore, Friebel et al. observed that the administration of Fxa inhibitors exerts pleiotropic properties, in addition to the well-known anticoagulatory ones, that lead to a decline in collagen intermediate plasma levels and to amelioration of the diastolic function in patients with HFpEF [41] (Figure 1). It has been observed that the degree of CCL is associated with LV filling pressures in patients with HFpEF and that the administration of torasemide, a pyridine-sulfonylurea class of loop diuretics, contributes to reducing the enhanced expression of LOX, without registering a decrease in the circulating collagen intermediates [42].

As mentioned, the other major mechanism is mediated by AGEs, complex and heterogeneous compounds, which increase with aging. The formation of these products drives protein towards the CCL through a reaction of the reducing oxo-group of sugars with a free ε-amino group of the protein such as the lysine or arginine amino groups [43]. Several studies, conducted in a variety of tissues, showed that the non-enzymatic addition of AGEs to the intramuscular connective tissue network reduces muscle and physical function with aging [21]. AGE formation is further fueled by metabolic derangements, such as diabetes mellitus, one of the main HFpEF driver comorbidities [44]. Non-enzymatic-induced CCL derives from the deposition of AGEs, the first step of the Maillard reaction [45]. AGEs contribute to decreased ventricular and vascular elasticity, leading to major stiffness and consequently to diastolic and vascular dysfunction. In addition, AGEs act indirectly on collagen formation and reduce nitric oxide bioavailability. AGEs are responsible for endothelial impairment by reducing vasodilation and increasing vasoconstriction through the production of endothelin-1. Inflammation fuels the deposition of AGEs, thus making sense of their augmented accumulation in HFpEF, characterized by a low-grade inflammatory milieu [46]. AGEs concur with HF also through interaction with their own receptors (RAGEs), which induce fibrosis due to the activation of the TGF-β signaling pathway [47]. Several compounds employed to treat a variety of cardiovascular diseases aim at decreasing the AGE levels. Statins, also known as hydroxymethylglutaryl-coenzyme A reductase inhibitors, are cholesterol-lowering medications that display pleiotropic effects, including an action towards AGEs. Among them, cerivastatin prevents the angiogenesis evoked by AGEs, interrupting the signaling pathway [48]. Moreover, among angiotensin-converting enzyme (AGE) inhibitors, in diabetic animals, ramipril seems to reduce the AGE accumulation [49]. A therapeutic consideration regarding the non-enzymatic CCL should not fail to mention sodium glucose cotransporter 2 inhibitors (SGLT2i), a novel class of glucose-lowering medications, recently approved for the treatment of HFrEF, even in the absence of diabetes [50]. The administration of SGLT2i seems to reduce the AGE-mediated consequences and the signaling pathway downstream interaction between AGEs and RAGEs in diabetic rats; furthermore, it appears to oppose the action of ROS, leading to an amelioration of the endothelial function [51]. Empagliflozin, one of the members of this novel class of anti-diabetic drugs, exhibits improved diastolic function in mice with a failing heart. However, it did not restore normal myocardial AGE levels [52]. Interestingly, the DELIVER trial proved that Dapagliflozin reduced the risk of worsening HF or cardiovascular death among patients with HFpEF [53]. Therefore, multiple pathways can be targeted to prevent or reduce AGEs, collagen accumulation and HFpEF progression. Nonetheless, in the last decades, spironolactone, and the other mineralocorticoid receptor (MR) blockers have raised great interest in HFpEF therapy armamentarium. The MR directly affects target gene expression, mainly of fluid, electrolyte, haemodynamic homeostasis and tissue remodelling, acting as a systemic modulator of extracellular matrix, inflammation and fibrosis. In fact, the pathophysiological over-activation of the MR leads to extensive remodelling of the cardio-renal system. In macrophages, MR activation promotes M1-phenotype differentiation, thus leading to galectin-3 secretion, fibroblast activation and deposition of fibrous tissue. In parallel, several major clinical trials with MR antagonists demonstrated a reduction in morbidity and mortality in patients with heart failure, even if the constraint of developing potentially life-threatening hyperkalaemia still represents an argument [54]. Nowadays, identifying specific targets for HFpEF could engender multiple benefits not only for the patient’s quality of life but also for the economic burden that this condition represents.

## 6. Conclusions

**Figure 2:**
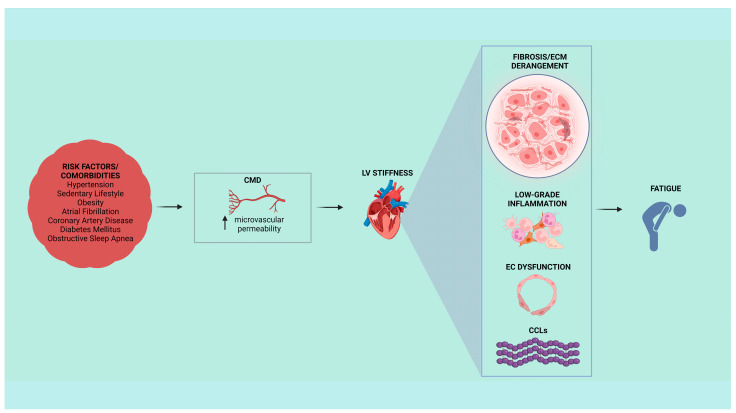
Cardiovascular risk factors and comorbidities could lead to typical symptoms of heart failure with preserved ejection fraction (HFpEF), such as fatigue. In HFpEF patients, left ventricular (LV) stiffness could be associated with coronary microvascular dysfunction (CMD) within a context of collagen-dependent fibrosis, extracellular matrix (ECM) derangement, low-grade inflammation, endothelial cell (EC) dysfunction, and genesis of new fibrils of collagen cross-links (CCLs).

Looking upon common “tales” between two, or more, clinical conditions, could unveil tangible goals and targets, improving the diagnostic and therapeutic management of patients with HFpEF [55]. Furthermore, in these patients, besides the presence of numerous risk factors and comorbidities, collagen deposition and fibrosis play a focal role in the rise and progression of adverse cardiac remodeling. Further studies are certainly needed to implement the knowledge on CMD involvement, ECM derangement as well as on low-grade inflammatory targets directly engage in the pro-fibrotic cascade, myocardial stiffness and impaired diastolic function. In light of the above, a better understanding of collagen alterations could certainly guide towards more effective diagnoses and novel therapeutic strategies for heterogeneous clinical conditions such as HFpEF (Figure 2).

## Data Availability

Not Applicable
